# Cross-border shopping: evidence from household transaction records

**DOI:** 10.1186/s41937-025-00141-w

**Published:** 2025-09-02

**Authors:** Frédéric Kluser

**Affiliations:** Department of Economics, University of Bern, Schanzeneckstrasse 1, CH-3001 Bern, Switzerland

**Keywords:** Cross-border shopping, Spatial consumption, R1, R2, L14

## Abstract

Cross-border shopping expands product variety and lowers prices for consumers in high-price countries, but it diminishes domestic tax revenues, reduces sales, and shifts demand away from local retailers. Exploiting Switzerland’s COVID-19-induced border closure as a natural experiment, I investigate the socioeconomic implications of cross-border shopping. Linking detailed grocery transaction records for 710,000 households to administrative data, I find that the border closure raises domestic grocery expenditures in border areas by an additional 10.4%. The benefits of cross-border shopping, however, are heterogeneous, and larger and lower-income households exhibit a particularly strong propensity to shop abroad. Based on these patterns, I estimate an annual loss of 1.5 billion Swiss francs in domestic grocery sales, equivalent to 3.8% of the total market.

## Introduction

Cross-border shopping is a significant economic activity in border regions, allowing consumers from high-price countries to access a wider array of goods at lower prices in nearby foreign markets. Although this raises purchasing power and choice near borders, it diminishes domestic tax revenues, shifts sales away from local retailers, and affects employment in border areas (see Leal et al., 2010, Knight and Schiff, 2012, or Baggs et al., 2018). As such, cross-border shopping shapes consumer behavior and contributes to unequal economic impacts across socioeconomic groups and regions.

This paper quantifies cross-border shopping in Switzerland and assesses heterogeneities across household backgrounds, offering new insights into cross-border shopping’s role in exacerbating or mitigating inequality. To accomplish this, I use Switzerland’s border closure as a natural experiment to investigate how consumers respond when their access to foreign markets is restricted. In 2020, many countries imposed travel restrictions to contain the spread of COVID-19, and on March 16, 2020, the Swiss government mandated the immediate closure of all national borders, along with domestic restaurants, bars, entertainment, and leisure facilities – exempting only supermarkets and pharmacies. This policy was upheld until June 2020.^1^

Among countries that implemented similar measures, Switzerland is particularly well-suited for studying cross-border shopping for two reasons. First, it borders countries where grocery prices are 28–39% lower, allowing Swiss households to purchase cheaper goods in Germany, Italy, Austria, or France.^2^ These countries share a common currency, simplifying price comparisons for Swiss shoppers.^3^ Second, the random timing of the Swiss border closure, combined with its stringent enforcement, drastically reduced cross-border shopping to almost zero (see Burstein et al., 2024), thereby providing a clean setting in which to measure its impact.

I identify the causal effect of Switzerland’s COVID-19 border closure on domestic grocery expenditures by comparing Swiss households living near the national border to Swiss households residing further inland, using a difference-in-differences framework. This approach captures the magnitude of cross-border shopping under normal conditions since the border closure forced consumers to shift previously foreign purchases to domestic retailers. To implement this analysis, I merge unique grocery data featuring the universe of customer-linked transactions from the country’s largest retailer for the year 2020 with individual-level administrative register records on labor market income, commuting behavior, and household characteristics for the entire Swiss population. The final dataset contains 31 million weekly shopping baskets for 710,000 households that I can uniquely link to residents in the administrative data, allowing me to investigate how cross-border shopping varies across socioeconomic backgrounds, location characteristics, and commuting behaviors.

My findings reveal that consumption patterns are remarkably persistent. I estimate a 10.4% increase in domestic grocery expenditures for border households, which disappears immediately once the border reopens, implying that cross-border shopping follows deeply rooted routines and is not easily altered by short-term shocks. Examining socioeconomic and regional heterogeneities, I observe the strongest responses among poorer and larger households and in areas where neighboring countries offer the most significant price discounts. The effect declines with distance, indicating a lower propensity to shop abroad among inland households. Synthesizing these insights, I calculate that cross-border shopping reduces Swiss grocery sales by 1.5 billion Swiss francs (1.64 billion USD on January 10, 2025), equivalent to 3.8% of the total Swiss market volume). Furthermore, I demonstrate that households commuting toward the border chain their work trips with cross-border shopping, offering new evidence on the role of commuting in shaping spatial shopping behaviors.

This paper contributes to two strands of the literature. First, it adds to prior work on cross-border shopping, which demonstrates that both consumers and retailers respond to changes in relative prices. For instance, a depreciation of the US dollar lowers the consumers’ propensity to cross into Canada (Chandra et al., 2014) while increasing US employment and the number of establishments near the border (Campbell and Lapham, 2004). Likewise, Asplund et al. (2007) document how cutting Danish spirits taxes diminishes alcohol sales in Sweden, and Baker et al. (2021) show that US customers avoid local sales taxes by shopping across state lines. Finally, Friberg et al. (2022) find that consumers further inland are more responsive to foreign price changes, whereas households near the border shop abroad in any case. This pattern implies that reliance on relative price changes alone may underestimate the true prevalence of cross-border shopping. Consequently, I adopt a different strategy by exploiting a policy that eliminates cross-border shopping entirely rather than simply modifying its relative prices.

Two other studies examine COVID-19-related border closures in a cross-border shopping context but address different questions. Friberg et al. (2024) analyzes the effect on Norwegian tax revenues, finding a 3.6$\%$ reduction nationwide and 27$\%$ in border regions, while Burstein et al. (2024) uses Nielsen data to show that cross-border shopping can reduce the cost of living by up to 14$\%$ in Swiss border regions. In contrast, my paper focuses on the customers’ behaviors and the rich heterogeneities therein. My data – unique transaction records linked to administrative data – is arguably better suited for this analysis than the Nielsen data, whose self-recorded reporting errors can be correlated with demographic variables (Einav et al., 2008).

Beyond cross-border shopping, this work links to the broader literature on spatial shopping and trip chaining, discussing how customers deliberately plan and adapt their grocery expenditures and shopping trips. For instance, Agarwal et al. (2020) shows that consumers travel shorter distances for goods with low storability. Furthermore, customers often combine multiple non-tradable service visits on a single trip, creating consumption externalities that explain one-third of the spatial concentration in non-tradable services (Oh and Seo, 2023), and Miyauchi et al. (2025) illustrate how modeling trip chaining is crucial for understanding the decline in demand for non-traded services during the COVID-19 era. Similarly, Relihan (2024) highlights how trip chaining can produce complex spatial equilibrium outcomes with distinct winners and losers. By demonstrating that households synchronize cross-border shopping with their daily commutes, my paper extends this line of research on spatial shopping and trip chaining.

The remainder of this paper is structured as follows: Section 2 introduces the grocery and administrative data. Section 3 discusses the empirical strategy, Section 4 presents my findings, and Section 5 concludes.

## Data

I combine unique transaction data from the largest Swiss retailer with administrative data from the Federal Statistical Office on a $100\times 100$ meter spatial resolution.

The grocery data provides information on all customer-linked purchases at the retailer *Migros* in 2020, collected through their loyalty program in which customers identify themselves at the checkout with their loyalty card to receive exclusive offers and discounts. This loyalty program captures 79% of the retailer’s total sales, and 2.4 million customers regularly participate (meaning, 33% of all Swiss residents above legal age). Furthermore, Migros charges the same prices throughout the country, independently of local purchasing power, wages, and costs. Hence, prices are not endogenously lower close to the border. Stores of similar size also generally offer similar goods, except for local products. The dataset contains the universe of 600 million customer-linked purchases for the year 2020 and provides information on individual customer characteristics, including the location of their residence coded on a grid of $100\times 100$ meter cells, their age, and household type.

I enrich the purchase data with comprehensive individual-level administrative records covering the entire Swiss population (8.7 million inhabitants in 2020). The *Population and Households Statistics* provides individual and household characteristics, reporting information on gender, age, household members, and precise residence location on the common $100\times 100$ meter grid. The *Old Age and Survivors Insurance* reports annual gross labor market income, which I adjust using the square root of household size.^4^ Finally, the administrative *Structural Surveys* capture education and commuting behavior for the sub-sample of customers who participate in the survey.^5^ Education levels are categorized as primary, secondary, or tertiary, while commuting behavior is defined by travel times in minutes, mode of transport, and workplace municipality.^6^

Both datasets measure addresses on a uniform spatial grid spanning 350,000 cells nationwide, with a mean population of 25 residents per cell. I merge the two datasets by identifying unique pairs of customers and residents using the common variables grid cell and age. This approach uniquely matches 1.3 million customers in the grocery data to a citizen and their household in the administrative data. Hence, I can match 54$\%$ of the 2.4 million regular customers, corresponding to 20$\%$ of all adult Swiss residents. The primary outcome of interest throughout this analysis is a household’s total grocery expenditures in a given week. I aggregate the individual shopping trips into weekly baskets and exclude customers spending less than 100 Swiss francs per capita a month before the shock (equivalent to 109.5 USD on January 10, 2025), since their baskets might not capture overall consumption accurately. This process generates a final data set including 710,000 households and 31 million weekly consumption baskets.^7^

**Table 1:** Household summary statistics

	Final Sample		Population	
	Mean	SD	Mean	SD
Age	56.69	15.85	54.88	17.50
Income (1,000 CHF)	101.33	130.82	88.71	119.76
Income Adjusted (1,000 CHF)	60.33	80.77	59.27	77.05
Time Home to Work (min.)	28.23	23.03	29.20	23.69
Time Home to Border (min.)	57.79	24.18	56.20	25.26
Time Work to Border (min.)	58.46	31.65	56.07	23.72

Panel b)	Pct.	N	Pct.	N
*Education*		474,918		2,311,582
Primary	9.6	45,810	13.3	308,462
Secondary	45.7	217,022	45.2	1,045,327
Tertiary	44.7	212,086	41.4	957,793
*Household Size*		709,152		3,987,616
1	18.8	133,558	36.9	1,471,897
2	36.1	256,202	32.8	1,306,437
3-4	36.4	258,368	24.9	991,644
5+	8.6	61,024	5.5	217,638
*Language*		708,512		3,972,031
German	76.5	541,943	71.4	2,835,480
French	20.1	142,376	24.1	958,419
Italian	3.4	24,193	4.5	178,132
*Population Density*		708,512		3,983,515
Urban	24.4	172,955	31.5	1,253,963
Suburban	57.7	408,755	51.1	2,036,939
Rural	17.9	126,802	17.4	692,613
*Nationality*		709,110		3,986,823
Swiss	85.9	609,311	74.0	2,949,241
European	12.2	86,797	22.4	892,265
African	0.4	3,084	1.1	43,841
Asian	0.9	6,431	1.6	65,232
N.American	0.1	952	0.3	12,263
S.American	0.4	2,535	0.6	23,981
*Commuting Mode*		97,593		445,740
Car	58.9	57,530	55.2	246,067
Public Transport	24.9	24,258	28.0	124,790
Other	16.2	15,805	16.8	74,883
Observations		709,152		3,987,616

*Notes:* The table shows summary statistics for the customers uniquely matched to the administrative data and compares them to all Swiss households. Income equals the total annual labor market income of a household in 1,000 Swiss Francs, and Income Adjusted adjusts for the square root of household size. All time variables measure the uncongested car travel time in minutes to the work location or the closest cross-border location. The variables *Commuting Mode and Education are only available for the sub-sample participating in the Structural Surveys*

**Table 2:** Transactions summary statistics

Group	Mean	SD	p50	p1	p99
*Weekly Grocery Purchases*					
Expenditures in Matched Sample	81.6	55.2	67.0	12.7	261.1
Expenditures in Full Sample	88.1	61.8	71.4	12.5	291.2
Shop Visits in Matched Sample	5.3	3.0	4.8	0.8	15.0
Shop Visits in Full Sample	6.1	3.5	5.5	0.9	17.5
*Expenditures by Age Group*					
20–34	70.8	43.8	60.4	11.8	206.5
35–44	93.7	59.5	80.3	13.2	268.7
45–54	98.1	64.7	82.5	13.7	294.7
55–64	83.9	55.2	70.6	13.2	263.8
65–74	69.7	43.9	59.1	12.5	213.8
75+	60.6	38.3	51.0	11.4	190.0
*Expenditures by Income Quintile*					
15,000–53,269	69.8	45.0	58.6	12.3	218.2
53,270–82,604	72.1	46.7	60.1	12.8	227.0
82,605–115,688	83.6	53.0	70.9	13.1	248.6
115,689–161,706	94.5	58.6	82.4	13.7	268.8
161,707+	103.8	67.1	89.8	13.3	306.5
*Expenditures by Education*					
Primary	62.0	41.1	51.1	11.4	205.3
Secondary	79.7	51.7	66.9	13.0	246.5
Tertiary	94.8	62.0	80.6	13.2	286.5
*Expenditures by Household Size*					
1	53.2	32.1	46.0	11.3	166.0
2	73.5	44.1	64.5	12.5	211.5
3–4	97.5	60.9	85.2	13.8	277.5
5+	109.8	73.1	93.7	14.0	325.3
Transactions in Matched Sampled	31,076,495				
Transactions in Full Sampled	69,401,621				

*Notes*: The table shows summary statistics for the weekly expenditures and trip frequency of customers that I can match to residents in the administrative data. I compare these statistics to the full transaction dataset, including the unmatched customers, and report statistics on sub-samples for the matched data. The statistics for the *Full Sample* apply the same sample selection criteria used for the matched sample to the 120 million weekly baskets (600 million shop visits) in the transaction data set

Table 1 presents summary statistics for the matched households and shows how many of them I observe for each variable. The average matched household reports an income of 60,000 Swiss francs (adjusted for the square root of household size), and the mean cardholder is 56.7 years old, while 44.7$\%$ possess a tertiary education, and 80$\%$ live in multi-person households. Comparisons with the entire administrative data confirm that the matched sample is broadly representative of the population. Further, Table 2 displays summary statistics for the transactions. The average household performs 5.3 transactions and spends 82 Swiss francs (90 USD on January 10, 2025) per week or 349 Swiss francs per month (382 USD on January 10, 2025). This corresponds to about 57$\%$ of the average household’s grocery expenditures based on administrative consumption surveys. Thus, my data captures a substantial part of grocery shopping. Examining subgroups shows that expenditures rise with household size and income, while they exhibit a hump shape across age. A comparison to the entire transaction data indicates that the matched customers’ shopping behavior is aligned with expenditures in the full sample.

Finally, I compute car travel times to foreign shopping locations and workplaces. To achieve this, I gather the location and Google review counts of all foreign supermarkets within 20 km of the Swiss border from *Google Maps*. This yields 117 cross-border locations and a total of 1,787 stores, of which 691 have at least 100 Google ratings. Table 6 lists the largest identified cross-border locations, showing the number of stores with at least 100 and 500 Google ratings. A municipality with many stores typically also has multiple larger stores with numerous Google reviews, and correlations among the population, the number of stores, and the number of stores with more than 100 and 500 Google ratings is high, lying between 0.83 and 0.92. As cross-border shoppers likely target larger stores, I define a cross-border location as a foreign municipality with at least three stores that have more than 100 Google ratings.^8^ Next, I retrieve the car travel time from every raster cell to all these locations from a national online mapping service (*search.ch*) and select the shortest trip for each cell. One-fifth of all households reach the closest cross-border location within a 30-min car drive, while the maximum distance is 3 h. Following the same approach, I compute distances to workplaces. Table 1 shows the average car travel time to the closest cross-border location (58 min) and to the work location (28 min). In total, 59$\%$ commute to work by car, while 25$\%$ use public transportation.

## Empirical strategy

**Fig. 1:**
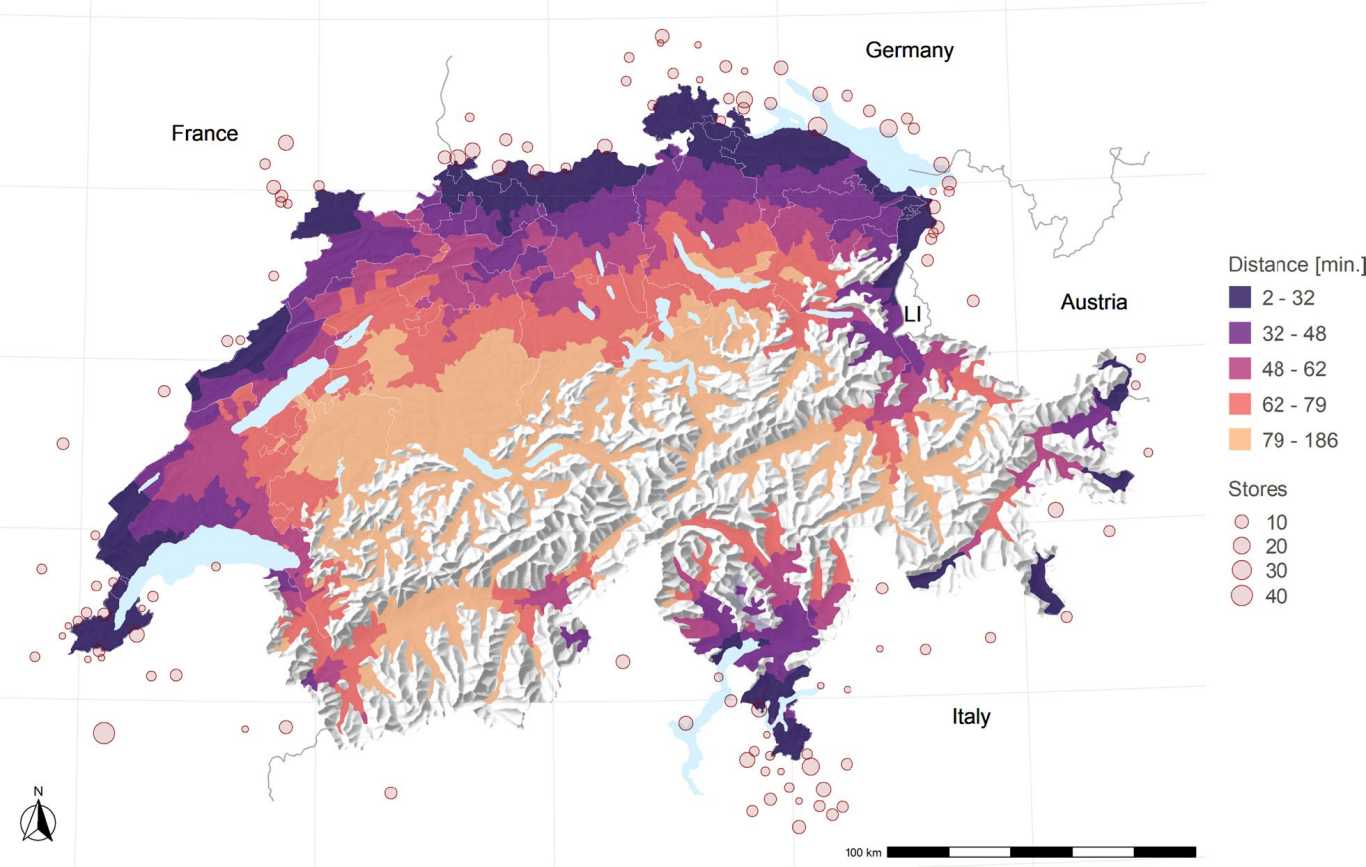
Distance to the Closest Cross-Border Shopping Location. *Notes*: The figure shows the quintiles of car driving times to the closest cross-border shopping location on the municipality level. The dots show all 117 cross-border locations within 20 kms of the Swiss border, and the dots’ size indicates the number of supermarkets at this location

I examine the impact of the border closure on household expenditures by comparing households residing within a half-hour car drive from a cross-border location (the first quintile) to those living far enough inland so that they rarely shop abroad. Hence, I define the control group as households living more than 80 car minutes away (the fifth quintile) and exclude all individuals residing within the doughnut area to ensure a clean control group. This yields a sample of roughly 125,000 treated and 135,000 control households.^9^ Figure 1 presents these travel distance bins to the closest foreign location across Switzerland. The figure further underscores the importance of explicitly using travel times to cross-border locations rather than the Euclidean distance to the border, given the dispersion of these shopping locations and the landscape’s morphology.

I use a difference-in-differences model to estimate the average treatment effect. Since all political regulations, grocery supply adaptations, and consumers’ behavioral changes affect both the treatment and control group, I attribute any deviation after the intervention to cross-border shopping. Nonetheless, the onset of COVID-19 potentially introduced significant behavioral changes not captured by time-constant fixed effects. Hence, if the COVID-19 pandemic affected treated and control units differently – beyond the border closure I exploit – my estimates could be biased if not carefully addressed. While time-varying covariates could account for these confounders, they introduce unintended identifying variation, even in a difference-in-differences setting with common treatment timing, and the resulting estimates are not ATTs (see Goodman-Bacon, 2021 and Sloczynski, 2022).

To address this challenge, I omit any control variables and instead provide a balance-check table that demonstrates the comparability of the treatment and control groups across key variables in Table 3, showing that the two groups did not diverge over time in any substantial way. In addition to these balance checks, I present in the Appendix (i) the distribution of travel times from home to work for both groups in 2019 and 2021 (Figure 4) and (ii) the number of COVID-19 cases as well as the mitigation policies’ stringency for the treatment and control groups throughout 2020 (Figure 5). Both plots confirm that any disparities in commuting trip duration, COVID-19 incidence, and governmental mitigation measures between these groups are minimal. Taken together, these analyses reinforce my key identification assumption, arguing that any observed differences in outcomes stem from the border closure itself rather than other changes during the pandemic.

**Table 3:** Balance checks

	Treatment Group				Control Group					
	2019		2021		2019		2021		$\Delta$ in p.p.	
	Count	%	Count	%	Count	%	Count	%	Coeff	SE
*Labor Market Status*										
Working	22,836	43.61	25,798	43.88	14,907	38.62	14,703	39.07	$-$0.2	(0.473)
Not Working	29,529	56.39	32,997	56.12	23,696	61.38	22,927	60.93	0.2	(0.473)
*Commuting*										
No Commuting	5,043	17.52	6,786	21.05	4,192	17.98	4,657	20.58	0.9	(0.481)
Within Mun.	6,943	24.12	7,317	22.70	5,537	23.75	5,245	23.18	$-$0.9	(0.531)
Within Canton	12,092	42.00	12,852	39.87	10,313	44.23	9,711	42.91	$-$0.8	(0.587)
Within CH	4,711	16.36	5,281	16.38	3,273	14.04	3,019	13.34	0.7	(0.443)
*Commuting Means*										
Walking / Bicycle	4,612	18.84	5,231	19.96	3,365	17.25	3,370	18.45	$-$0.1	(0.515)
Individual	11,810	48.23	13,562	51.74	11,431	58.61	11,031	60.38	1.7$$^{**}$$	(0.657)
Public	7,984	32.61	7,274	27.75	4,648	23.83	3,794	20.77	$$-$$1.8$$^{**}$$	(0.610)
*Weekly Two-Way Trips to Work*										
5+ trips	17,498	73.72	17,365	68.30	12,467	66.05	11,124	62.91	$$-$$2.3$$^{***}$$	(0.631)
Less than 5 trips	6,239	26.28	8,061	31.70	6,407	33.95	6,557	37.09	2.3$$^{***}$$	(0.631)
*Jobs*										
Managers	3,156	10.92	3,835	11.88	2,178	9.41	2,438	10.86	$-$0.5	(0.359)
Professionals	7,296	25.24	8,478	26.26	5,079	21.95	5,024	22.39	0.6	(0.515)
Technicians	5,317	18.40	5,837	18.08	4,061	17.55	3,970	17.69	$-$0.5	(0.479)
Clerical Support	3,833	13.26	3,934	12.18	2,734	11.82	2,626	11.70	$-$1.0$^{*}$	(0.398)
Service / Sales	3,726	12.89	4,182	12.95	3,605	15.58	3,290	14.66	1.0$^{*}$	(0.423)
Agriculture / Forestery	357	1.24	433	1.34	730	3.16	671	2.99	0.3	(0.186)
Craft / Trade Workers	2,474	8.56	2,668	8.26	2,511	10.85	2,245	10.00	0.5	(0.374)
Machine Operators	1,024	3.54	1,103	3.42	980	4.24	974	4.34	$-$0.2	(0.247)
Elementary Occupation	1,720	5.95	1,819	5.63	1,258	5.44	1,205	5.37	$-$0.2	(0.286)
*Income*										
Q1	520,210	27.64	518,353	27.27	384,961	25.00	396,237	25.41	$$-$$0.8$$^{***}$$	(0.069)
Q2	465,295	24.73	474,201	24.94	394,016	25.59	394,251	25.29	0.5$$^{***}$$	(0.066)
Q3	416,584	22.14	426,491	22.43	408,538	26.54	412,171	26.44	0.4$$^{***}$$	(0.065)
Q4	479,777	25.49	482,052	25.36	352,027	22.87	356,517	22.87	$-$0.1$^{*}$	(0.065)

*Notes:*This table provides balance checks between the control and treatment groups for the year before (2019) and after (2021) the border closure shock. The table uses individual-level data from the *Structural Surveys* for all variables, except income, which is derived from the *Old Age and Survivor’s Insurance*. The column labeled $\Delta$* in p.p.* shows the change in the difference of percentage shares between the groups, expressed in percentage points. Standard errors are calculated using 1,000 bootstrap replications

To estimate the average treatment effect, I follow Chen and Roth (2024) and Wooldridge (2023) and estimate a QMLE-Poisson model, as some households record zero expenditures in a given week^10^:

1 $$\begin{aligned} Y_{it} = \text {exp}\left( \alpha _i + \gamma _t + \sum _{\begin{array}{c} j= 1 \end{array}}^{52} \beta _j (D_{i} \times T_{j}) \right) \epsilon _{it}, \end{aligned}$$

where $Y_{it}$ denotes the grocery expenditures of household *i* in week $t \in 1, \dots , 52$. $\alpha _i$ and $\gamma _t$ are the household- and week-specific fixed effects, capturing unobserved heterogeneity. $D_{i}$ is an indicator variable that equals one if household *i* is in the treatment group, the dummy variables $T_{j}$ indicate the weeks of the year 2020, and $\beta _j$ are the associated pre- and post-treatment coefficients for each period *j*.

Treatment starts in week eleven, and I normalize coefficients to the pre-treatment average. I cluster standard errors at the zip code level and report in all tables and figures the transformed coefficients ${\hat{\beta }}_{ATT\%} = \exp ({\hat{\beta }} -1)$, which yields the average proportional treatment effects and allows me to interpret the coefficients as percentage changes. I compute the corresponding standard errors via the delta method.^11^

To examine heterogeneities in the treatment effect, I implement a static version of the model and interact the treatment indicator with a categorical variable $x_i$:

2 $$\begin{aligned} Y_{ikt} = \text {exp} \left( \alpha _i + \gamma _{tk} + \sum _{k \in \mathcal {K}} \beta _{k}( D_{i} \times Post_t \times x_{ik}) \right) \epsilon _{ikt}, \end{aligned}$$

where $Post_t = 1$ while the border is closed (meaning, $t \in [11, \dots , 24]$), and zero otherwise. $k \in \mathcal {K}$ indexes the individual categories of $x_i$, $x_{ik} = \mathbbm {1}(x_i = k)$, and $\beta _k$ is the average treatment effect for each group *k*. In this specification, the time dimension of the treatment effect collapses to a single post-treatment coefficient. I allow the time fixed effect to vary across the different groups *k* by including week-group fixed effects $\gamma _{tk}$ as the pandemic might affect the individual groups differently.

## Results and discussion

This section presents three sets of results. First, I analyze the average treatment effect of the border-closing policy on grocery expenditures over time. Second, I study the unequal response to the policy for different household backgrounds and commuting behaviors, highlighting which characteristics particularly benefit from cross-border shopping. Third, I examine the role of distance, assessing how actively customers shop abroad as travel costs increase. This approach furthermore allows for a discussion on potential spillovers to the control group. Finally, I connect these insights to calculate a measure for the annual reduction in domestic grocery sales due to cross-border shopping activity.

### Response to the COVID-19 border closure

**Fig. 2:**
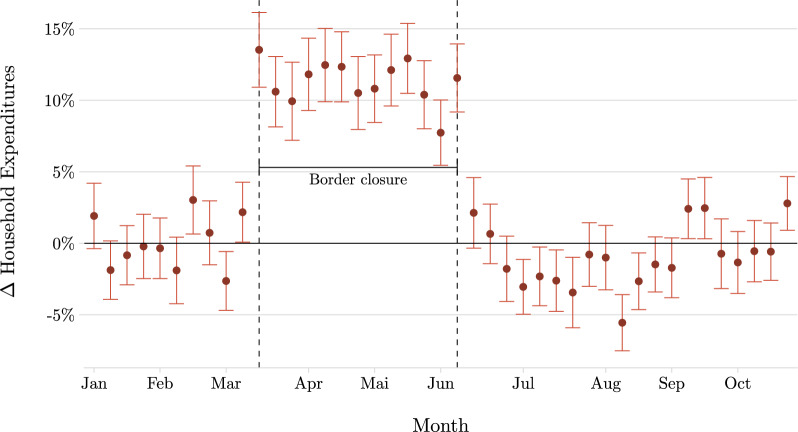
Dynamic Treatment Effects. *Notes*: The figure shows the border closure’s effect on household expenditures within a 30-minute car ride from a cross-border location compared to households living further away than 80 min. I indicate the period of border closure by vertical dashed lines. The regression estimates Equation (1) and uses 11.4 million observations. Coefficients are normalized to the pre-treatment periods’ average, and standard errors are clustered at the zip code level. Coefficients are exponentiated such that they equal proportional effects

Figure 2 shows the results for the dynamic difference-in-differences specified in Equation (1). The borders close in week 11 and reopen in week 25, and vertical dashed lines indicate both events. Aggregating all months during the border closure together, I find that the border closure temporarily raised domestic grocery expenditures significantly by 10.4%$$^{***}$$ (s.e.: 0.006) among households at the border relative to those residing further inland, with week-specific effects ranging from 8% to 14$$\%$$. These findings align with Burstein et al. (2024), who estimate that Swiss households close to the border spend roughly 8$$\%$$ of their expenditures abroad. Further, this expenditure shift is immediate and remains constant as long as the border is impassable. After the reopening, expenditures instantly drop to the previous level. Hence, although households in border regions temporarily increased their spending at domestic supermarkets, they did not permanently alter their cross-border shopping behavior through the border closure and fully reverted to their old patterns as soon as possible. This result suggests that cross-border shopping follows deeply rooted routines that withstand major temporary shocks.

One concern might be that consumers modified their shopping behavior before the actual introduction of pandemic restrictions, especially in strongly affected areas (for example, via stockpiling or by avoiding larger crowds). However, the insignificant pre-treatment coefficients in Figure 2 do not indicate any anticipation effects nor a potential violation of the parallel trend assumption between treated and control units, suggesting that households living in the border region and further inland did not react differently to the pandemic’s onset.

Furthermore, the estimates in Figure 2 remain unchanged under various robustness checks. For example, I (i) restrict the analysis to households who did not move during 2020, (ii) use the full sample of available transactions in the grocery data rather than focusing on the sub-sample of customers matched to residents in the administrative data, and (iii) redefine cross-border locations to only consider very large foreign stores that may be more attractive to travel to (see the corresponding event study plots in the Appendix, Figure 7, Figure 8, and Figure 9).

### The unequal benefits of cross-border shopping

In the following, I expand on these average treatment effects to study the potentially unequal benefits of cross-border shopping, studying heterogeneities across socioeconomic backgrounds, the closest neighboring country, and differences in commuting behavior.

#### The role of socioeconomic backgrounds

Consumers’ preferences for cross-border shopping might vary based on their socioeconomic background, resulting in unequal benefits of cross-border shopping. Hence, I analyze treatment effect heterogeneities for different household characteristics in the rich administrative register data and Table 4 reports the estimation results of Equation (2) for the variables household size, age, income, education, nationality, and neighboring country. The table also includes p-values, testing the treatment effects’ equality over the different groups (meaning, the null hypothesis is $\beta _k = \beta \; \forall k$).

**Table 4:** Treatment Effects by Socioeconomic Subgroups

*Dep. Variable:* Household Expenditures					
a) Household Size		b) Age		c) Income	
Group	Coeff	Group	Coeff	Group	Coeff
1	0.059$$^{***}$$	20–34	0.124$$^{***}$$	Q1	0.138$$^{***}$$
	(0.006)		(0.010)		(0.008)
2	0.098$$^{***}$$	35–44	0.139$$^{***}$$	Q2	0.124$$^{***}$$
	(0.007)		(0.009)		(0.007)
3-4	0.130$$^{***}$$	45–54	0.133$$^{***}$$	Q3	0.107$$^{***}$$
	(0.008)		(0.008)		(0.008)
$$5+$$	0.140$$^{***}$$	55–64	0.119$$^{***}$$	Q4	0.100$$^{***}$$
	(0.010)		(0.008)		(0.011)
		65–74	0.128$$^{***}$$	Q5	0.091$$^{***}$$
			(0.009)		(0.015)
		75+	0.109$$^{***}$$		
			(0.010)		
p-value	0.000	p-value	0.013	p-value	0.000
n	6,029,696	n	6,029,045	n	4,382,236

d) Education		e) Nationality		f) Country	
Group	Coeff	Group	Coeff	Group	Coeff
Primary	0.135$$^{***}$$	Swiss	0.100$$^{***}$$	AT	0.078$$^{***}$$
	(0.010)		(0.006)		(0.014)
Secondary	0.102$$^{***}$$	European	0.152$$^{***}$$	GER	0.102$$^{***}$$
	(0.006)		(0.012)		(0.008)
Tertiary	0.105$$^{***}$$	African	0.206$$^{***}$$	FR	0.113$$^{***}$$
	(0.008)		(0.039)		(0.010)
		Asian	0.150$$^{***}$$	IT	0.361$$^{***}$$
			(0.026)		(0.041)
		N.American	0.168$$^{**}$$		
			(0.061)		
		S.American	0.129$$^{**}$$		
			(0.044)		
p-value	0.003	p-value	0.000	p-value	0.000
n	3,950,694	n	6,029,226	n	5,849,849

*Notes*: The table shows the border closure’s average treatment effect on household expenditures within a 30-minute car ride from a cross-border location compared to households living further away than 80 min, separately for different household characteristics. These characteristics include *household size*, *age* of the registered cardholder, household *income* adjusted by the square root of household size, the highest *education* in the household, the cardholders’ *nationality*, and the *country* of their closest cross-border shopping location. The regression estimates Equation (2), standard errors are clustered at the zip code level, and the reported p-values test the equality of all coefficients. Coefficients are exponentiated such that they equal proportional effects

First, I find that the effect increases with household size. While a treated one-person household raises expenditures by only 5.9$\%$ in response to the border closure, expenditures rise by 9.8$\%$ for two-person households and by 13$\%$ for households with at least three members. Thus, larger households engage in more cross-border shopping. Traveling abroad to shop at lower prices is particularly tempting if one buys large quantities regularly, since it increases the trip’s savings while the trip’s traveling costs are fixed. Such economies of scale likely explain this finding, as the summary statistics in Table 2 show that larger households spend more money on groceries overall and consume larger quantities, making cross-border shopping more attractive for them.

Second, I find heterogeneous effects across ages in the response to the border closure. The estimated effect lies around 13% for young households between age 20 and 44 and diminishes gradually as households become older. Nonetheless, even retired households after age 65 show a relatively high response of roughly 12%, despite their markedly lower total expenditures (see Table 2). This result might be driven by the sharp decline in their income after retirement, which motivates them to shop abroad at lower prices. Furthermore, they presumably face lower opportunity costs. Note that this heterogeneity may reflect either age or cohort effects, given that the short sample period does not permit disentangling them.

Third, I examine income. On the one hand, one should anticipate households with a lower income to engage in more cross-border shopping given their higher import elasticities (see Auer et al. 2024) and the larger share of their income dedicated to groceries. For instance, households in the top income quintile allocate only 1.7$\%$ of their revenue to groceries, whereas lower income households in the bottom quintile spend 4.6$\%$. On the other hand, less affluent households might be less mobile, partly due to lower car ownership rates. While 90$\%$ of high-income households in Switzerland (top quintile) own a car, this holds for only 77$\%$ of low-income households, according to the Federal Statistical Office. Likewise, lower income households travel, on average, fewer kilometers per day (30.2 kms vs. 40.8 kms). The results in panel c) show that the first line of arguments dominates the narrative: the treatment effect declines from 13.8$\%$ for the lowest earning quintile to 9.1$\%$ for the highest-earning households. Hence, although traveling costs are relatively high for many of them, lower income households still engage in more cross-border shopping activity.

Fourth, we might expect higher educated customers to respond more strongly to the border closure due to their broader knowledge and enhanced access to information, which facilitates strategically optimizing their consumption. However, these households typically face fewer budget constraints, making cost savings less essential. Consequently, I find that households with at least one tertiary-educated member raise their expenditures by 10.5%, while the effect is notably higher (13.5%) among households without tertiary education, where financial considerations likely dominate.

As a final source of heterogeneity, I investigate the role of neighboring countries and their grocery prices. Panel f) of Table 4 displays the spatial variation in the effect by estimating heterogeneous treatment effects for the four neighboring countries: Austria, Germany, France, and Italy.^12^ The results indicate a substantial effect for households living closest to Italy (36%), compared to smaller effects for those near France (11%), Germany (10%), and Austria (8%). To examine the influence of price differences behind these findings, I re-estimate Equation 2 by interacting the treatment with national price level indices for food and non-alcoholic beverages. Table 7 reports national price level indices averaged over 2015–2020 for major product categories, along with the percentage by which these products are less expensive relative to Switzerland. While all product categories are uniformly cheaper abroad, the relative price differences vary across countries and categories. Although these national averages offer only a rough proxy for the prices Swiss households actually encounter – given that foreign retailers may set higher prices near the Swiss border and regional price variations exist within each country – the results provide valuable insights. The analysis shows a significant positive effect of log prices in the closest neighboring country on domestic household expenditures during the border closure, with an estimated coefficient of 0.0211$$^{***}$$ (SE: 0.001). Nevertheless, these results should be interpreted with caution, as they rely on the assumption that national price averages adequately reflect border prices and cross-border shopping dynamics.

In summary, cross-border shopping benefits exhibit strong heterogeneity by socioeconomic and spatial factors. Larger and lower income households respond more, reflecting economies of scale and budget constraints, while proximity to Italy yields the largest effect, in line with higher price differentials. These patterns underscore how income, mobility, and geography determine cross-border shopping behavior.

#### Commuting behavior and trip chaining

Furthermore, I analyze the interaction of cross-border shopping behavior and daily work trips, as commuting may be a significant driver of a household’s shopping decisions (see, for example, Miyauchi et al. (2025)). Cross-border shopping and commuting might interact in two ways. First, households can combine commuting and shopping by trip chaining if their workplace is closer to the border than their home. Second, frequent commuting trips to work may alter a household’s perception of distance and traveling costs, potentially influencing their likelihood of traveling abroad, even if their workplace lies far away from the border. Hence, I use Equation (2) to estimate the treatment effect separately for households commuting either from home (i) toward foreign shopping locations or (ii) farther inland, away from cross-border locations. I focus on households that live 20 to 35 min from the border and commute by car.

**Table 5:** Treatment Effect for Different Commuting Behaviors

	*Dep. Var:* Household Expenditures		
	Commute	Commute	
$\Delta$ Border Access	Toward Border	Away f. Border	p-value
Treat $$\times$$ 5-15 min	0.142$$^{***}$$	0.093$$^{***}$$	0.316
	(0.018)	(0.018)	
Treat $$\times$$ 15-25 min	0.156$$^{**}$$	0.101$$^{***}$$	0.035
	(0.053)	(0.025)	
n	339,012		

*Notes:* The table shows the border closure’s average treatment effect on household expenditures within a 30-min car ride from a cross-border location compared to households living further away than 80 min for different household commuting trips. These trips include commutes by car for 5-15 min and 15-25 min, either toward the national border (bringing the commuter closer to a cross-border location) or further away from the border in comparison with the household’s home. The regression estimates Equation (2) and standard errors are clustered at the zip code level. Coefficients are exponentiated such that they equal proportional effects

Table 5 shows the estimation results. On the one hand, households whose commute takes them 5 to 15 min closer to the border increase their cross-border shopping by 14.2$\%$ in response to the border closure. For households whose workplace is 15-25 min closer to a cross-border location, I estimate an effect of 15.6$\%$. On the other hand, I observe lower effects of 9.3$\%$ and 10.1$\%$, respectively, for households commuting away from the border. Together, these two observations strongly suggest that households combine work commutes with cross-border shopping trips via trip chaining. This reinforces evidence on strategic trip chaining in Miyauchi et al. (2025), Oh and Seo (2023), and Relihan (2024) within the context of cross-border shopping activity.

### The role of distance

**Fig. 3:**
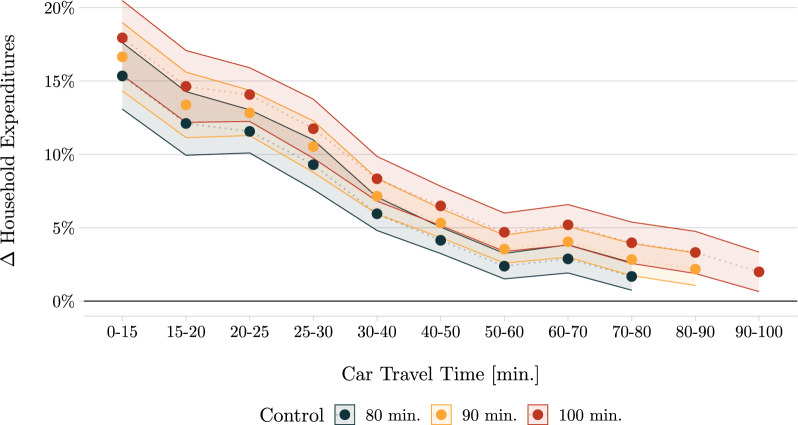
Decay of the Treatment Effect. *Notes*: The figure shows the border closure’s average treatment effect on household expenditures for households living within a certain distance bin. I compare these treated units to households living further away than 80, 90, and 100 min from the closest cross-border location, respectively. Standard errors are clustered at the zip code level. The regressions estimate Equation (2) and all use roughly 16.5 million observations. Coefficients are exponentiated such that they equal proportional effects

**Fig. 4:**
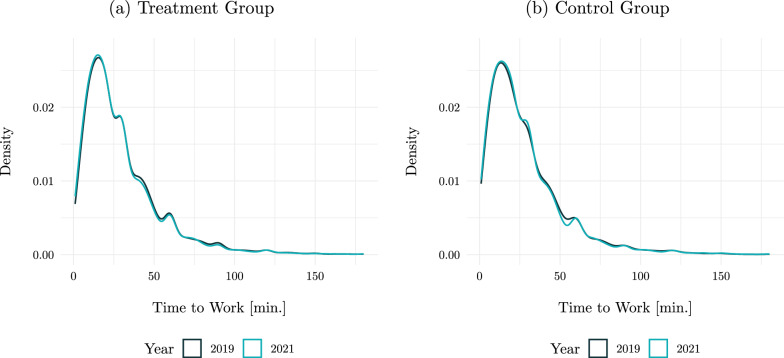
Time to work.*Notes:* The figure shows the density of car travel times to workplaces in minutes for the treatment group (Figure 4a) and the control group (Figure 4b) before (2019) and after (2021) the treatment

**Fig. 5:**
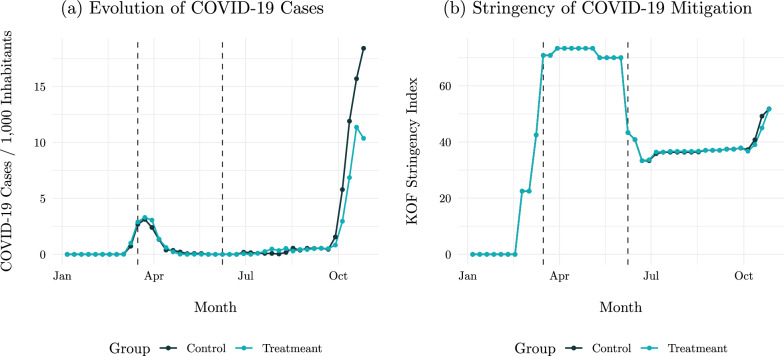
COVID-19 and mitigation measures. Figue 5a shows the evolution of the cantonal COVID-19 cases per 1,000 inhabitants for the treatment and control group over time. Figure 5b shows the KOF Stringency Index for the mitigation measures’ stringency in Switzerland, again separated by the two groups

**Fig. 7:**
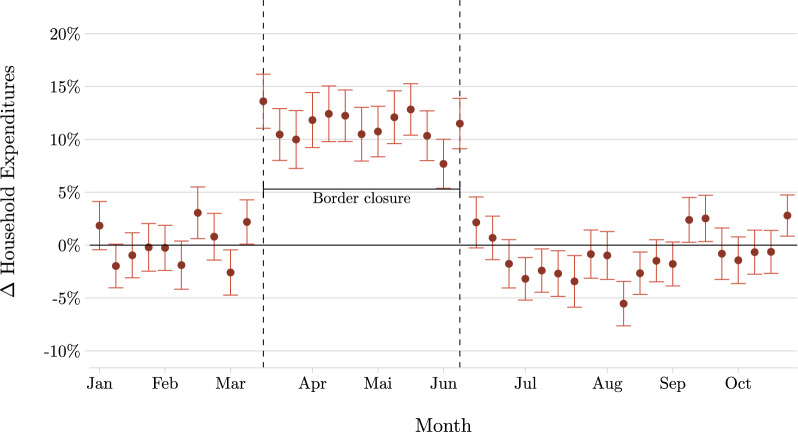
Robustness of the dynamic treatment effects: excluding movers in 2020. *Notes*: The figure shows the border closure’s effect on household expenditures within a 30-minute car ride from a cross-border location compared to households living further away than 80 min. I indicate the period of border closure by vertical dashed lines. The regression estimates Equation (1) and uses all the 10.9 million observations for households who did not move in 2020. Coefficients are normalized to the pre-treatment periods’ average, and standard errors are clustered at the zip code level. Coefficients are exponentiated such that they equal proportional effects

**Fig. 8:**
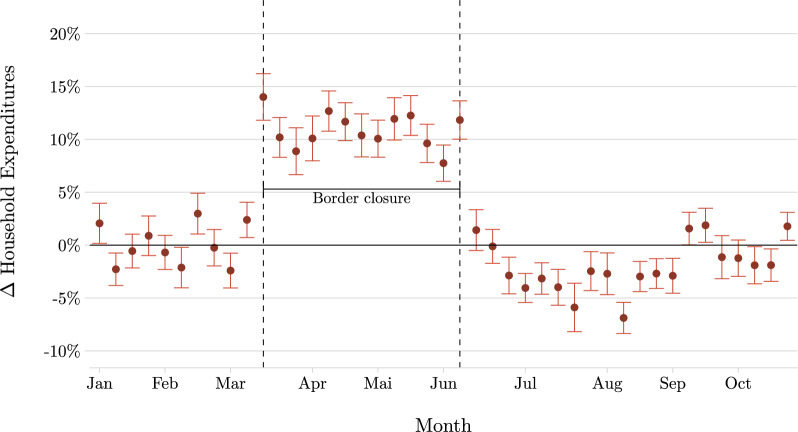
Robustness of the dynamic treatment effects: the full grocery transaction data. *Notes*: The figure shows the border closure’s effect on household expenditures within a 30-minute car ride from a cross-border location compared to households living further away than 80 min. I indicate the period of border closure by vertical dashed lines. The regression estimates Equation (1) and uses all the 23.8 million observations in the full grocery transaction data. Coefficients are normalized to the pre-treatment periods’ average, and standard errors are clustered at the zip code level. Coefficients are exponentiated such that they equal proportional effects

**Fig. 9:**
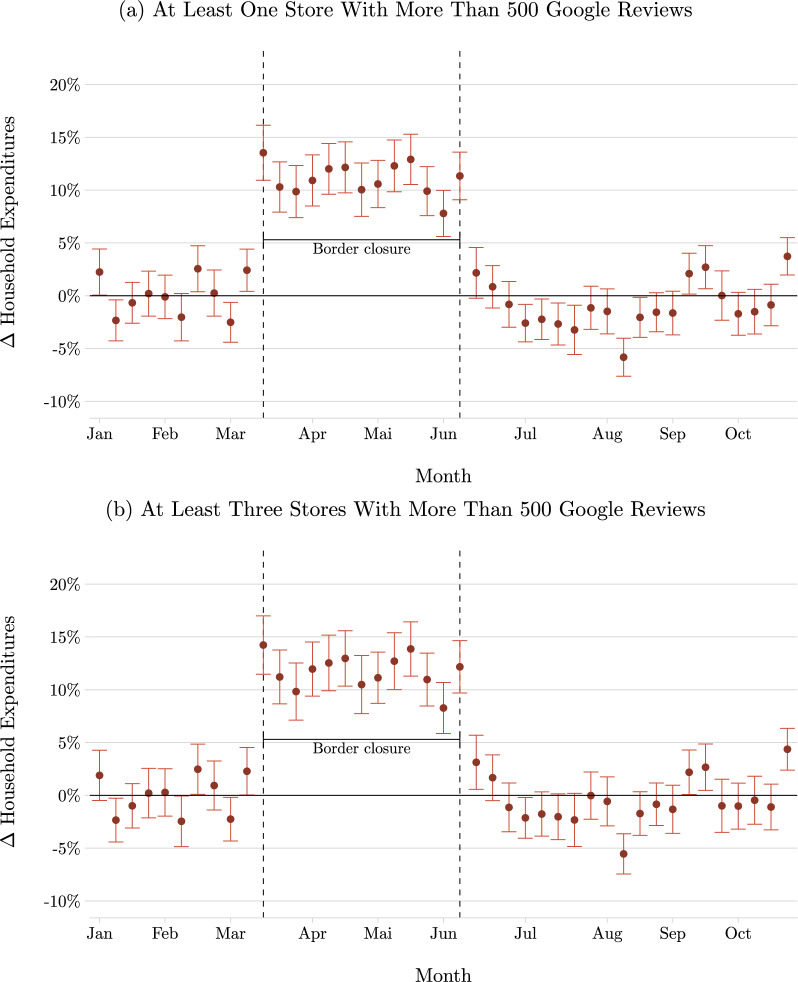
Robustness of the dynamic treatment effects: different definitions of cross-border locations. *Notes*Figure 9a shows the border closure’s effect on household expenditures within a 30-minute car ride from a cross-border location compared to households living further away than 80 min. I consider all cross-border locations with at least one store with more than 500 Google reviews. In comparison, Figure 9b shows the same results but considers locations with at least three stores with more than 500 Google reviews. The regressions both estimate Equation (1) and use 12.0 and 12.5 million observations, respectively. Standard errors are clustered at the zip code level. Coefficients are exponentiated such that they equal proportional effects

Throughout the previous sections, I choose a doughnut–specification defining control households as those living at least an 80-min car drive from the closest cross-border shopping location. Yet, choosing the radius of the inner doughnut defines which households are excluded from my analysis and features a trade-off between (i) ensuring that the treatment does not affect the control units and (ii) maintaining a sizeable and representative control group. If households living 80 min from a cross-border location are still impacted, my results should be regarded as lower bounds. To investigate this, I now consider larger doughnut areas. Figure 3 compares the distance decay function for my preferred specification to two alternative approaches using control households with at least a 90-min and 100-min trip to the closest cross-border location.

Focusing on the preferred specification of 80 min in Figure 3, I find that households living within a 15-min radius of the closest cross-border destination increase their expenditures by 16% during the border closure. This effect declines linearly up to a distance of 50 min before flattening out, yet remains significant beyond 80 min. Note that these distances may underestimate the actual travel distance since customers might opt to shop at other foreign stores farther away rather than at the closest location.

**Fig. 10:**
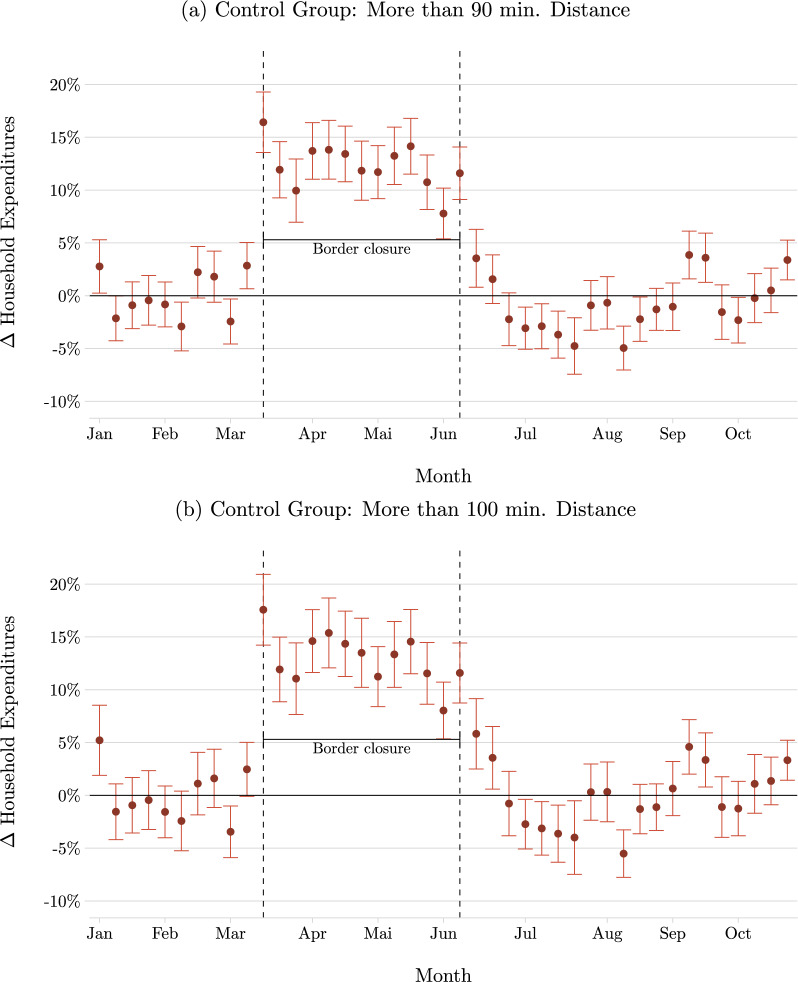
Robustness of the dynamic treatment effects: different control distance.*Notes*: Figure 10a shows the border closure’s effect on household expenditures within a 30-minute car ride from a cross-border location compared to households living further away than 90 min. I indicate the period of border closure by vertical dashed lines. The regression estimates Equation (1) and uses 8.3 million observations. Figure 10b also estimates Equation (1) for a distance of 100 min using 6.7 million observations. Coefficients are normalized to the pre-treatment periods’ average, and standard errors are clustered at the zip code level. Coefficients are exponentiated such that they equal proportional effects

Comparing to the alternative specifications indicates that some control units in my baseline results are likely still influenced by the border closure, given the significant coefficient for the last distance bin. Since the alternative approaches consistently report higher point estimates, I probably underestimate the true effect. On the other hand, the size of the control group shrinks significantly from 135,000 to 64,000 and 27,000 households under stricter definitions of control units. To manage this trade-off, I choose the most conservative approach and present in the paper all estimates with a control group consisting of households living 80 min from the border. In the Appendix, Figure 10 displays the event study results for a 90-min and 100-min control group, while Table 9 and Table 10 replicate the previous results for a control distance of 100 min and reaffirm that my conclusions and arguments remain qualitatively the same.

### Quantify the total effect of cross-border shopping on domestic sales

To quantify the overall domestic sales lost due to cross-border shopping, I estimate the following equation:

3 $$\begin{aligned} Y_{it} = \text {exp} \left( \alpha _i + \gamma _{t} + \beta (D_i \times Post_t \times {\textbf {X}}_i) \right) \epsilon _{it}, \end{aligned}$$

where the interacted controls ${\textbf {X}}_i$ include all variables used in the heterogeneity analysis (income bins, education levels, age bins, household size, and neighboring country dummies). This specification allows me to compute an estimate for the sales lost as follows. First, I subtract for the treated households the fitted values of expenditures ${\hat{Y}}_{it}$ from the predicted values under the counterfactual scenario without a border closure policy (i.e., $D_i = 0$). Second, I aggregate these differences to derive an annual total value, weighting them by the inverse of the number of customers in my sample relative to the number of residents in the customers’ municipality. This rescaling assumes that the unobserved customers are comparable to the observed ones, which appears plausible based on prior evidence regarding my data’s representativeness. Finally, I account for the fact that we observe only a sub-sample of each household’s total expenditures. Drawing on Kluser and Pons (2024), who show that my data covers a representative 65% of the average household’s total expenditures, I scale up my result accordingly. This procedure yields an estimated total loss of grocery food sales caused by outgoing cross-border shoppers of 1.5 billion Swiss francs, corresponding to 3.8% of the total Swiss market volume. This estimate, based on the temporarily imposed autarky, is notably lower than the survey-based 3.3 billion Swiss food expenditures abroad in Rudolph et al. (2022). Under additional assumptions, this suggests that cross-border shopping under the tax-free allowance of 300 CHF accounts for 230 million CHF in foregone VAT revenues per year (0.9% of total VAT revenues). With the 2024 reduction of the allowance to 150 CHF, the estimated VAT loss falls to roughly 72 million CHF (0.3%). These estimates combine my own data with key results from Rudolph et al. (2022) and rely on the assumption that domestic and cross-border expenditures are log-normally distributed. Future research could relax these assumptions and study the households’ price sensitivity as well as compliance with declaration rules, which is likely incomplete and may change with policy.

## Conclusion

This paper exploits the Swiss COVID-19-related border closure as a natural experiment to study the heterogeneous benefits of cross-border shopping. I document that cross-border shopping is both widespread and persistent in Switzerland and that domestic sales would be 10.4$\%$ higher in border regions without it. I then uncover heterogeneities: larger, lower income, less educated, and younger households engage in more cross-border shopping, and the response is larger where the neighboring country’s grocery price indices are relatively low. I also present novel evidence that households commuting toward the border combine their trip to work with shopping abroad, whereas commuting away from the border has no effect.

These findings carry important implications. First, the unequal benefits from cross-border shopping may inform normative assessments of the optimal spatial supermarket allocation, highlighting the greater sensitivity of households with a lower willingness to travel. Second, the results can support policies seeking to mitigate any negative externalities of cross-border shopping for domestic employment, consumption, sales, and tax collection (see, for example, Leal et al. (2010); Knight and Schiff (2012), and Baggs et al. (2018)).

Ultimately, while numerous spatial models in economics integrate agents’ commutes and a broad empirical literature identifies patterns in commuting behavior, mobility for shopping remains understudied. An important exception is Miyauchi et al. (2025), who jointly model commuting and shopping trips in a quantitative spatial framework. However, they cannot observe expenditures, focusing instead on the trips themselves–thus missing the intensive margin of spatial shopping. Future work could bridge this gap by incorporating the empirical insights on shopping presented here, resulting in a richer depiction of the spatial equilibrium and enabling more credible counterfactual analyses.

## Figures and Tables

**Fig. 6:**
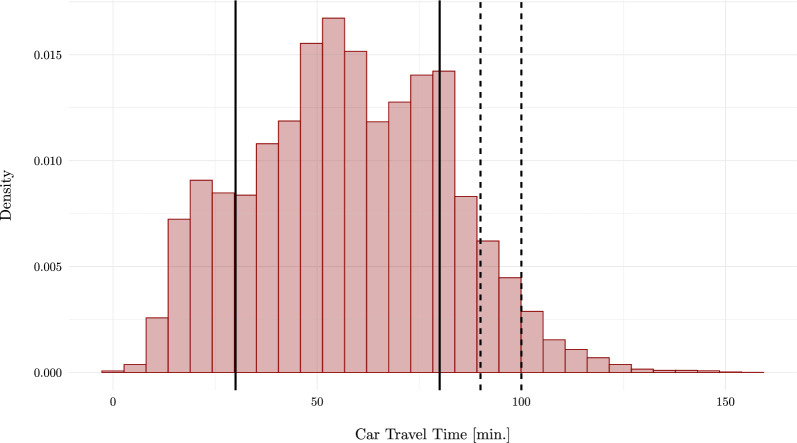
Distribution of travel times. *Notes*: The figure shows the distribution of car travel times from a household’s home to the closest cross-border shopping location. The sub-samples of control units used in the different robustness checks of the dynamic results are marked by vertical dashed lines

## Data Availability

The data used for this article from Migros is proprietary and not publicly available. The data from the Federal Statistical Office and the Central Compensation Office can be requested at the respective offices through the data linkages for third parties process.

## Appendix

See Figures 4, 5, 6, 7, 8, 9, and 10 and Tables 6, 7, 8, 9, and 10

**Table 6:** Cross-border locations

	Location	Country	Population	Number of Stores			Rank		
				*Google Reviews*			*Google Reviews*		
				-	100	500	-	100	500
1	Annecy	FR	131,766	79	29	11	1	1	3
2	Como	IT	84,808	76	21	14	2	4	1
3	Konstanz	GER	84,446	71	29	14	3	1	1
4	Singen	GER	48,033	50	18	10	4	5	4
5	Annemasse	FR	36,582	49	13	5	5	13	15
6	Aosta	IT	34,052	47	7	3	6	30	34
7	Livigno	IT	6,363	47	14	5	6	12	15
8	Varese	IT	80,588	46	15	7	8	8	8
9	Friedrichshafen	GER	61,561	45	23	10	9	3	4
10	Sondrio	IT	21,457	40	3	1	10	67	67
11	Cantù	IT	40,031	39	12	6	11	16	10
12	Belfort	FR	45,458	37	15	4	12	8	22
13	Lindau	GER	25,547	36	15	9	13	8	6
14	Domodossola	IT	17,930	35	11	4	14	18	22
15	Lörrach	GER	49,295	33	15	7	15	8	8
16	Weil am Rhein	GER	30,009	31	18	9	16	5	6
17	Saronno	IT	39,332	30	9	6	17	24	10
18	Waldshut-Tiengen	GER	24,067	30	13	6	17	13	10
19	Stockach	GER	17,118	29	11	5	19	18	15
20	Radolfzell	GER	31,582	28	7	4	20	30	22
21	Überlingen	GER	22,684	27	13	4	21	13	22
22	Rheinfelden	GER	32,919	26	16	5	22	7	15
23	Bad Säckingen	GER	17,510	25	11	4	23	18	22
24	Bregenz	AT	29,806	25	12	5	23	16	15
25	Montbéliard	FR	25,806	25	10	3	23	22	34
$$\dots$$									
*Overall*									
117			1,980,614	1,787	691	304			

*Notes*: The table shows the 25 largest cross-border locations for grocery shopping. *Number of Stores* counts the municipality’s stores for a given minimum of Google reviews, while *Rank* ranks the locations according to the number of stores. All store locations are scraped from Google Maps

**Table 7:** Prices in neighboring countries, 2015–2020

Category	Austria		France		Germany		Italy	
	PI	vs. CH	PI	vs. CH	PI	vs. CH	PI	vs. CH
Clothing and Footwear	102.83	-20%	105.53	-18%	98.80	-23%	100.52	-22%
Consumer Goods	106.37	-20%	107.02	-20%	103.12	-23%	105.18	-21%
Food and non-Alcoholic Beverages	120.47	-28%	112.38	-33%	102.52	-39%	109.30	-35%
Households Appliances	95.08	-21%	105.37	-12%	101.18	-16%	101.50	-15%
Recreation and Culture	113.27	-26%	107.28	-30%	104.57	-32%	100.10	-35%
Restaurants and Hotels	108.67	-35%	119.73	-28%	105.88	-36%	104.02	-38%

*Notes*: The table shows prices in neighboring EU countries averaged over the six years before and during the first wave of the COVID-19 pandemic, 2015–2020. Prices are shown as price indices (PI) for different product categories and relative to the category’s price index in Switzerland. In each year, the EU27 average is set to 100

**Table 8:** Treatment effects by socioeconomic subgroups (with a 90 min. control group)

*Dep. Variable:* Household expenditures					
a) Household Size		b) Age		c) Income	
Group	Coeff	Group	Coeff	Group	Coeff
1	0.071$$^{***}$$	20–34	0.128$$^{***}$$	Q1	0.141$$^{***}$$
	(0.008)		(0.011)		(0.008)
2	0.113$$^{***}$$	35–44	0.157$$^{***}$$	Q2	0.127$$^{***}$$
	(0.007)		(0.010)		(0.008)
3-4	0.140$$^{***}$$	45–54	0.143$$^{***}$$	Q3	0.119$$^{***}$$
	(0.008)		(0.009)		(0.009)
$$5+$$	0.150$$^{***}$$	55–64	0.133$$^{***}$$	Q4	0.115$$^{***}$$
	(0.011)		(0.009)		(0.014)
		65–74	0.151$$^{***}$$	Q5	0.139$$^{***}$$
			(0.011)		(0.020)
		75+	0.120$$^{***}$$		
			(0.011)		
p-value	0.000	p-value	0.002	p-value	0.095
n	4,374,752	n	4,374,357	n	3,149,267

d) Education		e) Nationality		f) Country	
Group	Coeff	Group	Coeff	Group	Coeff
Primary	0.146$$^{***}$$	Swiss	0.110$$^{***}$$	AT	0.087$$^{**}$$
	(0.012)		(0.006)		(0.030)
Secondary	0.102$$^{***}$$	European	0.167$$^{***}$$	GER	0.117$$^{***}$$
	(0.007)		(0.013)		(0.008)
Tertiary	0.119$$^{***}$$	African	0.184$$^{***}$$	FR	0.107$$^{***}$$
	(0.009)		(0.050)		(0.011)
		Asian	0.158$$^{***}$$	IT	0.403$$^{***}$$
			(0.035)		(0.042)
		N.American	0.240$$^{**}$$		
			(0.095)		
		S.American	0.154$$^{**}$$		
			(0.055)		
p-value	0.000	p-value	0.000	p-value	0.000
n	2,878,473	n	4,374,446	n	4,195,995

**Table 9:** Treatment Effects by Socioeconomic Subgroups (With a 100 min. Control Group)

*Dep. Variable:* Household expenditures					
(a) Household size		(b) Age		(c) Income	
Group	Coeff	Group	Coeff	Group	Coeff
1	0.086$$^{***}$$	20–34	0.135$$^{***}$$	Q1	0.133$$^{***}$$
	(0.011)		(0.016)		(0.010)
2	0.113$$^{***}$$	35–44	0.165$$^{***}$$	Q2	0.132$$^{***}$$
	(0.008)		(0.013)		(0.011)
3-4	0.146$$^{***}$$	45–54	0.152$$^{***}$$	Q3	0.129$$^{***}$$
	(0.010)		(0.011)		(0.010)
$$5+$$	0.159$$^{***}$$	55–64	0.137$$^{***}$$	Q4	0.132$$^{***}$$
	(0.014)		(0.010)		(0.018)
		65–74	0.142$$^{***}$$	Q5	0.124$$^{***}$$
			(0.011)		
		75+	0.128$$^{***}$$		
			(0.013)		
p-value	0.000	p-value	0.149	p-value	0.994
n	3,510,431	n	3,510,060	n	2,496,237

d) Education		e) Nationality		f) Country	
Group	Coeff	Group	Coeff	Group	Coeff
Primary	0.136$$^{***}$$	Swiss	0.119$$^{***}$$	AT	0.090$$^{*}$$
	(0.016)		(0.008)		(0.037)
Secondary	0.104$$^{***}$$	European	0.169$$^{***}$$	GER	0.117$$^{***}$$
	(0.009)		(0.017)		(0.010)
Tertiary	0.128$$^{***}$$	African	0.189$$^{**}$$	FR	0.132$$^{***}$$
	(0.012)		(0.075)		(0.016)
		Asian	0.148$$^{***}$$	IT	0.432$$^{***}$$
			(0.043)		(0.041)
		N.American	0.173$^{*}$		
			(0.091)		
		S.American	0.140		
			(0.078)		
p-value	0.009	p-value	0.023	p-value	0.000
n	2,343,137	n	3,510,171	n	3,331,950

*Notes*: The table shows the border closure’s average treatment effect on household expenditures within a 30-minute car ride from a cross-border location compared to households living further away than 100 min, separately for different household characteristics. These characteristics include *household size*, *age* of the registered cardholder, household *income* adjusted by the square root of household size, the highest *education* in the household, the cardholders’ *nationality*, and the *country* of their closest cross-border shopping location. The regression estimates Equation (2), standard errors are clustered at the zip code level, and the reported p-values test the equality of all coefficients. Coefficients are exponentiated such that they equal proportional effects

**Table 10:** Treatment effect for different commuting behaviors (with a 100 min. control group)

	*Dep. Var:* Household Expenditures		
	Commute	Commute	
$\Delta$ Border Access	Toward Border	Away f. Border	p-value
Treat $$\times$$ 5-15 min	0.160$$^{***}$$	0.110$$^{***}$$	0.320
	(0.022)	(0.022)	
Treat $$\times$$ 15-25 min	0.174$$^{***}$$	0.118$$^{***}$$	0.033
	(0.055)	(0.028)	
n	163,627		

*Notes*: The table shows the border closure’s average treatment effect on household expenditures within a 30-minute car ride from a cross-border location compared to households living further away than 100 min for different household commuting trips. These trips include commutes by car for 5-15 min and 15-25 min, either toward the national border (bringing the commuter closer to a cross-border location) or further away from the border in comparison to the household’s home. The regression estimates Equation (2) and standard errors are clustered at the zip code level. Coefficients are exponentiated such that they equal proportional effects

## Footnotes

1 The borders to Liechtenstein remained open, although crossings between Liechtenstein and Germany or Austria were restricted. Nonetheless, the 370,000 workers commuting from neighboring countries into Switzerland and 29,000 Swiss residents working abroad were permitted to cross for work-related reasons.

2 Imports into Switzerland are VAT-exempt for a total value below 300 Swiss francs, with additional limits on items like meat and tobacco. Switzerland also borders Liechtenstein (population 40,000), which uses the Swiss franc and has almost identical grocery prices.

3 The CHF/EUR exchange rate remained stable throughout this period.

4 The calculation is $\text {income adjusted} = \frac{\text {income total}}{\sqrt{\#\text {household members}}}$, where I consider all household members, including small children. The adjustment follows one of the equivalence scales suggested by the OECD. I compute *income total* as the household’s annual income by summing the income of all household members.

5 This representative cross-sectional survey selects 200,000 people above age 15 every year. Customers can be selected repeatedly, and participation is mandatory. To measure education, I use the highest-reported education between 2010 and 2021 and exclude customers younger than 30 to capture students. For commuting, I only use the surveys since 2018 as workplaces are less stable than education.

6 Primary (or compulsory) education ends at the latest after around eleven mandatory years of school (including kindergarten). Customers who completed high school or an upper-secondary specialized school have a secondary education. Completing any degree at a university, university of applied sciences, or university of teacher education results in a tertiary degree.

7 See Kluser and Pons (2024) and Kluser et al. (2024) for additional information on the two data sources, the matching procedure, and the representativeness of the matched households for the general population.

8 My results are robust if I define cross-border locations alternatively as (i) locations with at least one store with 500 Google reviews or as (ii) locations with at least three stores with 500 Google reviews.

9 If a fraction of control units still reacted to the border closure because the distance to the border is not large enough, my estimates should be regarded as a lower bound. I will address this further in Section 4.3, showing that my results are robust if I use alternative comparison distances of 90 or 100 min.

10 Chen and Roth (2024) show that using a linear model with $log(Y+1)$ as a dependent variable does not allow interpreting the coefficients as percentage changes. Instead, estimating a QMLE-Poisson model and reporting the transformed coefficients ${\hat{\beta }}_{ATT\%} = exp({\hat{\beta }} -1)$ leads to the desired result.

11 Alternatively, I derive standard errors from 1,000 clustered bootstrap replications. The bootstrapped standard errors are quantitatively very similar.

12 For this spatial heterogeneity analysis, I use week fixed effects, as opposed to week-group fixed effects employed for socioeconomic variables.
